# Unusual Acute, Massive Lower Gastrointestinal Bleeding Secondary to a Proximal Jejunal Gastrointestinal Stromal Tumor: A Case Report

**DOI:** 10.7759/cureus.35287

**Published:** 2023-02-22

**Authors:** Ahlam A Alsulaiman, Mohammed N AlAli, Mohamed S Essa, Ossama Alamri, Abdullah M Albdah, Khaled S Ahmad

**Affiliations:** 1 Faculty of Medicine, College of Medicine, Vision College, Riyadh, SAU; 2 Department of Surgery, Prince Mohammed bin Abdulaziz Hospital, Ministry of Health (MOH), Riyadh, SAU

**Keywords:** gist, small intestine, laparoscopic resection, jejunal gist, massive lower gi bleeding

## Abstract

The most common primary non-epithelial neoplasms of the gastrointestinal (GI) tract are gastrointestinal stromal tumors (GISTs). Ten percent (10%) of GISTs arise from the jejunum. Usually, patients complain of abdominal discomfort, but they may present with complications such as intestinal obstruction or bleeding. This report describes a 35-year-old male who presented with unusually massive, acute lower GI bleeding. After resuscitation and investigations (including a contrast-enhanced computed tomography of the abdomen and lower GI endoscopy), the patient underwent diagnostic laparoscopy and bowel resection of the affected section with anastomosis, and he had an eventful postoperative course. Studies suggest that GI bleeding in GIST occurs due to the ulceration and necrosis of the overlying mucosa caused by the pressure effect of the mass. Small-bowel GISTs are categorized based on their size. Many guidelines have advocated conservative management for small GISTs (<2 cm) that are in the jejunum. This patient has a rare case of a small jejunal GIST with a rare presentation of massive lower GI bleeding. A multidisciplinary approach is useful in managing such cases, and our case showed that laparoscopic intervention is a feasible option in a stable patient with massive lower GI bleeding.

## Introduction

A gastrointestinal stromal tumor (GIST) is one of the mesenchymal tumors that arise from any part of the gastrointestinal (GI) tract [1-2]. The most common anatomical site for GIST is the stomach (two-thirds of the cases) and then the small bowel, mainly the duodenum (one-fourth) [1-4]. Rarely, GIST may develop from extra-gastrointestinal sites such as the omentum and retroperitoneum [1-2,5]. In decreasing order of frequency, GISTs rank fourth after carcinoids, gastrointestinal adenocarcinomas, and lymphomas [6]. The overall incidence of GISTs is extremely low (1 in 100,000), and jejunal GISTs represent 0.1-3% of gastrointestinal tumors, which is extremely rare [1-4]. GIST occurs commonly in middle-aged and older populations and is rare before the age of 40 [2,4,7]. The majority of cases are sporadic while approximately 5% are syndrome-related [3,5]. The common presentation of jejunal GIST is abdominal discomfort [2,5]. But, gastrointestinal bleeding may present uncommonly due to mucosal ulceration [2,4]. Therefore, we are reporting a case of a small jejunal GIST with an extremely uncommon presentation of acute massive lower gastrointestinal bleeding.

On January 20, 2023, this article was uploaded to quillbot.com for preprinting.

## Case presentation

**Figure 1:**
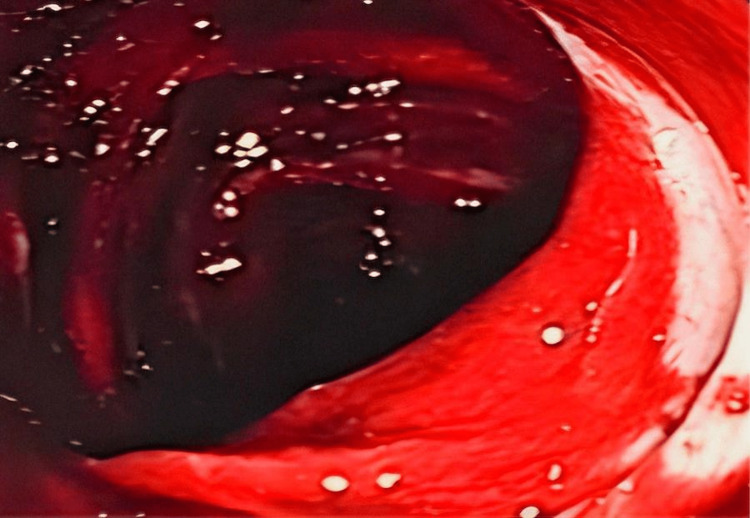
Endoscopic visualization, which reveals a colon filled with blood

A 35-year-old male, not known to have any chronic diseases, presented to the emergency room with acute massive lower gastrointestinal bleeding. There were no other symptoms or bleeding risk factors, and he was not on any medications. On physical examination, he was fully conscious and oriented but appeared pale. His heart rate was 130 beats per minute, and his blood pressure was 130/80 millimeters of mercury. The abdomen exam was unremarkable, whereas the rectal examination revealed hematochezia. A nasogastric tube was inserted, and the output was consistent with gastric contents. Laboratory tests revealed a leukocyte count of 11.4 x 10^9/L (reference range: 4.5-11.0 x 10^9/L), a hemoglobin level of 8 g/dL (reference range: 13.2-16.6 g/dL), and a hematocrit level of 25 (reference range: 41%-50%). The platelet and the international normalized ratio (INR) levels were normal; the rest of the blood tests were unremarkable. After resuscitation (1.5 liters of ringer lactate were given, followed by two units of packed red blood cells, with monitoring of the vital signs, urine output, and venous blood gases), he was stabilized and transferred to the endoscopy suite for upper and lower endoscopies. The upper gastrointestinal endoscopy was unremarkable while the lower gastrointestinal endoscopy showed that the colon was filled with blood coming from the distal ileum through the ileocecal valve (Figure 1).

**Figure 2:**
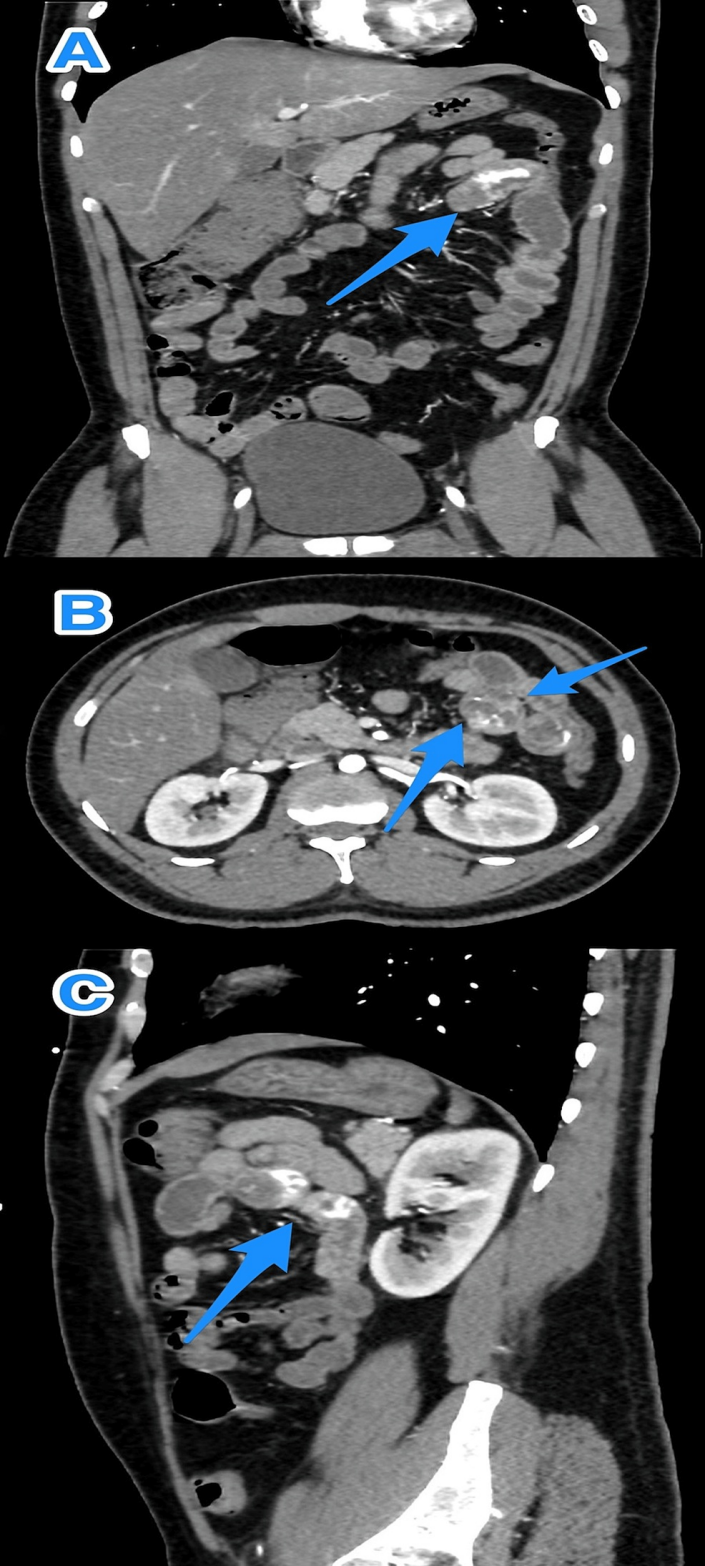
A multiview CT abdomen scan (A-C) reveals a proximal jejunal mass with extravasation (arrows) CT: computerized tomography

**Figure 3:**
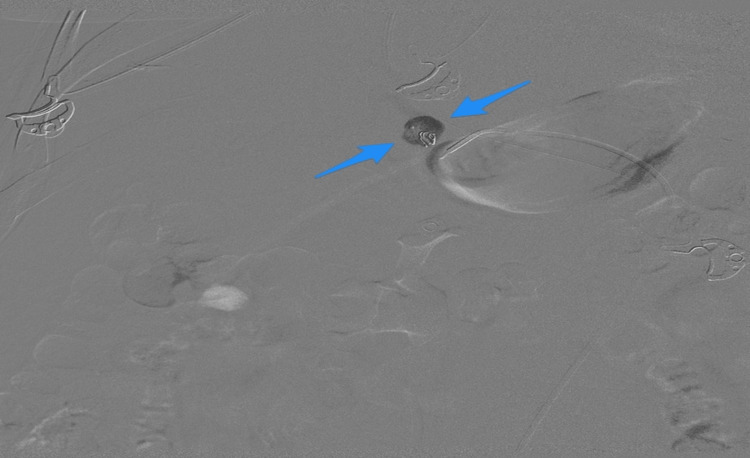
SMA angiography reveals extravasation (arrows) SMA: superior mesenteric artery

**Figure 4:**
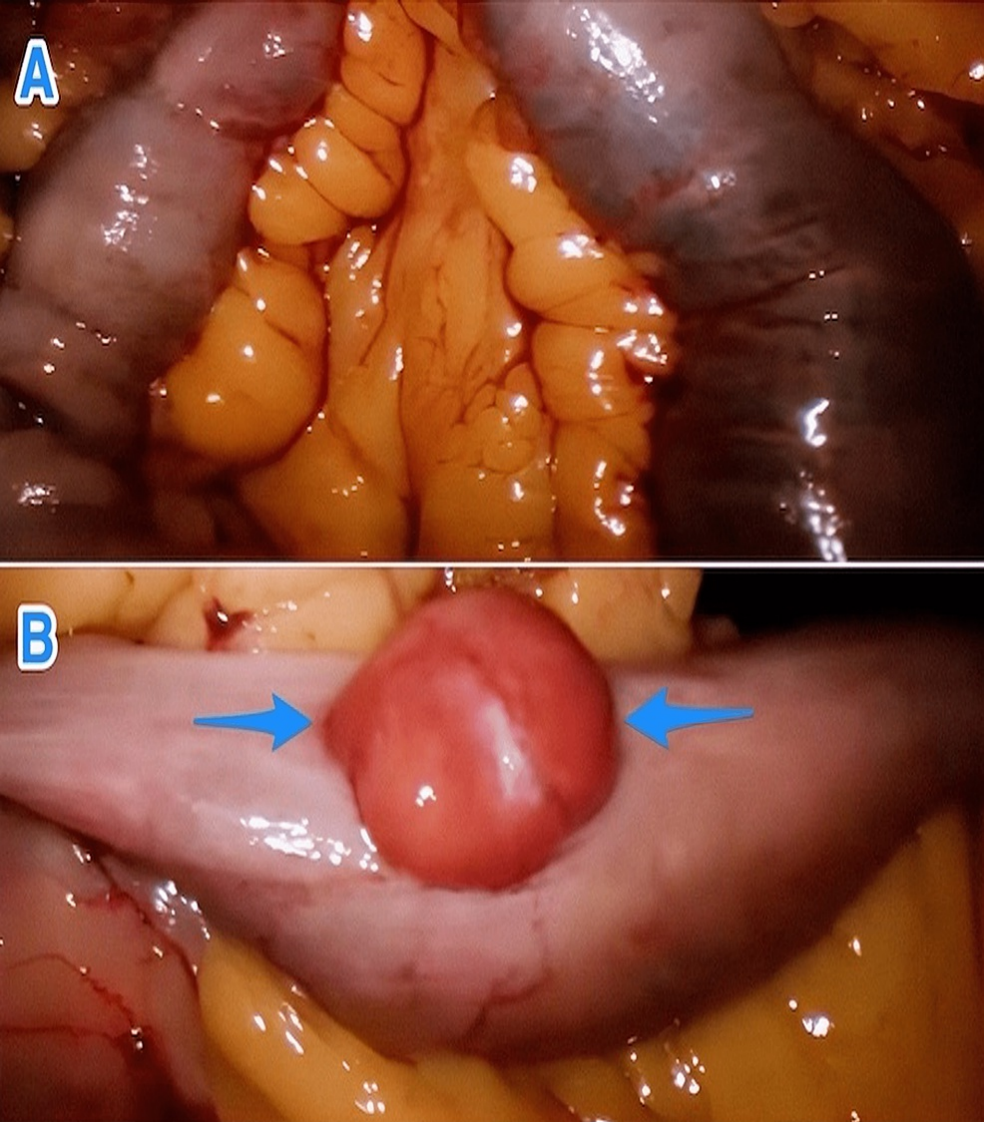
Intraoperative views of a blood-filled small bowel (A) and a GIST in the proximal jejunum (arrows) (B) GIST: gastrointestinal stromal tumor

So, a contrast-enhanced computed tomography (CECT) of the abdomen was performed and showed active extravasation of contrast from a 2 x 2 cm mass in the proximal jejunum (Figure 2). Consequently, superior mesenteric artery angiography was performed and confirmed the CT findings. So, embolization of the bleeder was attempted but was unsuccessful, as the interventional radiologist could not embolize and stop the bleeding (Figure 3). Therefore, after approximately five to seven hours of presenting to the emergency department, the patient underwent emergency surgery. The small bowel was filled with blood to allow a laparoscopic approach, the abdomen was explored and showed a small bowel full of blood and the previously mentioned mass on the antimesenteric border of the proximal jejunum (Figure 4). So, segmental resection with primary anastomosis was performed.

The patient had an uneventful postoperative course and was discharged on the sixth postoperative day. The histopathological report of the resected jejunal segment demonstrated a well-defined submucosal tumor with ulcerated mucosa extending to the muscular and serosal layers. Spindle cells arranged in fascicles and separated by fibrous septa comprised the tumor. The cells' cytoplasm was eosinophilic, and their nuclei were oval and spindle-shaped. Immunohistochemistry established the presence of CD117. As the tumor was classified as having a low risk of recurrence (G1), measuring 2 cm x 3 cm, with a mitotic index of 4 mitosis/high power fields (HPF), adjuvant therapy was not indicated (Stage I, T2N0M0). The patient was on regular follow-up visits (2, 6, and 12 weeks) at the outpatient clinic. He was doing well, with no active issues. The patient was advised to undergo a long-term follow-up consisting of a history, physical exam, and computed tomography (CT) of the abdomen every six months for three to five years.

## Discussion

The interstitial cells of Cajal (ICCs), which are considered the GI pacemaker, form the cellular origin of GISTs and regulate GI motility. Some GISTs are discovered accidentally, either by endoscopy or cross-sectional imaging performed for other purposes. GISTs could be associated with nonspecific symptoms that are highly dependent on the location of the tumor, for example, early satiety and fullness, or with complications, such as ulceration, bleeding, or an increase in size leading to pain and obstruction. Further, there are paraneoplastic syndromes reported in a few GIST patients. It has been hypothesized that tumor invasion of the mucosa layer results in GIST-related gastrointestinal bleeding [1-2,4-5,8].

The preferred imaging modality to assess most abdominal masses is contrast-enhanced computed tomography (CECT) [3]. GISTs are characterized, based on their size, as small (less than 5 cm), intermediate (5-10 cm), and large GISTs (more than 10 cm) [9]. Endoscopy remains the first line of investigation for gastrointestinal bleeding, but a single modality cannot locate whether the mass is extra-mural or intra-mural. Endoscopic ultrasound (EUS) identifies the original layer of a tumor and enables tissue biopsies for histopathological studies if indicated [3]. Therefore, in most cases, endoscopic ultrasound is performed along with endoscopy to locate the origin. In GISTs, positron emission tomography with fluorodeoxyglucose (FDG-PET) allows the early recognition of the tumor's response to tyrosine kinase inhibitors (TKIs), such as imatinib, which is may be required as neoadjuvant treatment [3]. A biopsy is not indicated in a resectable mass if it's highly suggestive of GIST (location, endoscopic appearance, and characteristics of the tumor on a sonographic and computed tomography scan) [3]. On trans-abdominal sonography, GISTs are characterized as a homogenously hypoechoic mass in close proximity to the gastrointestinal tract in addition to necrosis, cystic changes, and hemorrhages [3,9]. Further, the majority of GISTs detected by CECT are well-defined extraluminal or intramural masses with size-dependent attenuation [3,9]. If indicated, endoscopic ultrasound (EUS) -guided biopsy is superior to image-guided percutaneous biopsy [3]. GISTs are categorized into three types based on their histological features: spindle cell type (70%) as in our case, epitheliod type (20%), and mixed type (10%) [3]. GISTs are distinguished by the expression of the KIT protein, the mutations in the KIT gene (4 q 11-12), and the platelet-derived growth factor receptor alpha (PDGFRA) gene [3]. Moreover, CD34 expression was observed in two-thirds of GIST cases [10].

Even with the success of imatinib therapy and newer tyrosine kinase inhibitors [3,4], surgical resection with negative microscopic margins is the best treatment option for localized GIST. Laparoscopic resection is technically feasible for GISTs located in the stomach and small intestine measuring less than 5 cm [3,11]. En-bloc resection of the involved organs, such as the spleen, colon, or liver, could be adequate for locally advanced tumors. Lymphadenectomy is not indicated due to the rarity of lymph node metastasis [12]. Because tumor rupture is associated with a poorer prognosis due to peritoneal seeding [12], every effort should be made to prevent tumor rupture. In most cases with GISTs, metastatic spread occurs late in the course of the disease, commonly to the liver and peritoneum. Lymph node metastasis is very rare (0-8% of patients) [13].

Patients with disseminated disease may be candidates for palliative resection because long-term survival has been reported in some cases [12,14]. However, contradictory evidence exists regarding the relationship between gastrointestinal bleeding and the risk of recurrence in GISTs, and the prognosis is relatively poor compared to that of patients without bleeding [1,2,4]. Overall, the prognosis for tumors of the small intestine, colon, rectum, and mesentery is less favorable than that of the stomach [1,5]. The daily dose of neoadjuvant imatinib is 400 mg/day; unless a mutation in exon 9 is present, the dose will be higher (800 mg/day, 400 mg given twice daily) [3,15]. Due to resistance, patients with neurofibromatosis (NF)-associated gastrointestinal stromal tumors, succinate dehydrogenase (SDH)-deficient gastrointestinal stromal tumors, and platelet-derived growth factor receptor (PDGFRA) D842V mutant gastrointestinal stromal tumors can not receive adjuvant imatinib therapy [3,15]. The optimal duration of TKIs as neoadjuvant therapy is not yet defined [3,16].

## Conclusions

A multidisciplinary approach is useful for managing rare conditions and atypical presentations, such as jejunal GIST, with acute massive lower gastrointestinal bleeding. Endoscopy and computed tomography angiography are helpful in the diagnosis and localization of lower gastrointestinal bleeding such as identifying submucosal gastrointestinal tumors. Laparoscopic intervention is feasible in stable patients with a GIST of the small intestine.
